# RTAS-Net: A ResNet-transformer-ASPP semantic segmentation network for remote sensing images

**DOI:** 10.1371/journal.pone.0343729

**Published:** 2026-04-02

**Authors:** Ziheng Wang, Yang Li, Kejia Ma

**Affiliations:** School of Electric and Information Engineering, Changchun University of Science and Technology, Changchun, Jilin, China; Shandong Agricultural University, CHINA

## Abstract

Semantic segmentation of remote sensing images faces pronounced scale variation and complex class-wise spatial distributions, which often lead to semantic discontinuity in large regions and the loss of fine details for small objects. To address these issues, this paper proposes a U-Net–based remote sensing semantic segmentation network termed RTAS-Net (ResNet–Transformer–ASPP Segmentation Network), which enhances feature representation through a collaborative design of multi-scale context aggregation, fine-scale reinforcement, and local-to-global modeling. At the high-semantic level, the network incorporates ASPP to aggregate multi-scale contextual information and enlarge the effective receptive field, while integrating the window-based self-attention mechanism of Swin Transformer to model cross-region dependencies, thereby improving semantic consistency over large-scale areas. At high-resolution skip connections, a lightweight mini-ASPP is embedded to reinforce and pre-fuse fine-scale neighborhood information, and MobileViT is introduced to strengthen local texture and fine-grained structural representations, thus enhancing the recognition and boundary delineation of small objects. Rather than a simple stacking of modules, RTAS-Net achieves unified modeling of global semantics and local details through coordinated cross-level pathways. Experimental results on the ISPRS Potsdam, Vaihingen and LoveDA datasets demonstrate that the proposed method achieves consistent improvements in mIoU, mF1, and OA, and further provides a comprehensive analysis of parameter scale and inference efficiency, validating its effectiveness and practical applicability.

## Introduction

With the rapid advancement of remote sensing technology and the widespread availability of high-resolution aerial imagery, the automatic analysis and interpretation of remote sensing images have become critical tasks in various domains, including Geographic Information Systems (GIS) [1–3], urban planning [4,5], and environmental monitoring [6–8]. Traditional image processing techniques, such as image classification, image fusion, and edge detection, have achieved some success in the early stages of development. However, their performance remains limited when addressing the unique challenges of remote sensing imagery, such as significant variations in object scale, blurred object boundaries, and complex distributions of semantic categories.

Remote sensing images often exhibit extreme variations in object scales, ranging from large-scale structures such as roads and buildings to fine-grained elements like vehicles and street furniture. This diversity poses a significant challenge for single-scale feature extraction strategies, which struggle to balance global context with local detail. To address this issue, DeepLab [9] and its ASPP module employ dilated convolutions in parallel to capture multi-scale contextual information and effectively enlarge the receptive field. PSPNet [10] utilizes pyramid pooling to aggregate global context, while HRNet [11] maintains high-resolution feature streams to improve detail modeling. More recent approaches such as BAFNet [12] and SegDesicNet [13] incorporate long-range attention mechanisms and spatial embeddings to further expand the scale-aware receptive range. However, these methods often suffer from high computational overhead, rigid fusion architectures, or insufficient detail representation. To alleviate the problems of blurred boundaries and loss of structural detail in remote sensing images, encoder–decoder frameworks like U-Net [14] and SegNet [15] introduce skip connections to recover spatial resolution. DeepLabv3+ [16] enhances boundary reconstruction by integrating shallow features into the decoder. MobileViT [17] combines the spatial sensitivity of CNNs with the contextual modeling power of Transformers [18], thereby improving the representation of small objects and fine-grained structures. DFAFNet [19] employs attention-guided upsampling to refine edge modeling, while BAFNet [12] uses a dual-path design to integrate both local and long-range information for boundary-aware segmentation. Nevertheless, these methods still face limitations in fine edge modeling due to shallow semantic representations and structural redundancy. In the context of complex semantic category distributions and overlapping inter-class relationships in remote sensing scenes, Swin Transformer [20] and MobileViT [17] enhance the model‘s ability to capture long-range dependencies and delineate class boundaries through windowed attention and localized embedding mechanisms. Lite Transformer [21] achieves contextual modeling with lower computational cost. Modules such as SE [22], CBAM [23], SCSE [24], and CoordAttention [25] improve the response to salient features from channel, spatial, and positional perspectives. LMFNet [26] uses a shared-weight multi-branch architecture for cross-modal alignment, while SegDesicNet [13] incorporates geographic coordinate embeddings to enhance spatial semantic consistency. Despite these advancements, challenges remain in terms of receptive field coverage, depth of modeling, and structural generalizability, leaving room for further improvement.

To address the challenges outlined above, we propose RTAS-Net (ResNet-Transformer-ASPP Segmentation Network), a remote sensing semantic segmentation network designed to support multi-scale modeling, enhance boundary awareness, and improve global semantic understanding. Built upon the U-Net framework, RTAS-Net introduces a design philosophy of structural complementarity and path-wise collaboration. By integrating multiple functional modules, the model achieves improved adaptability to scale variations, enhanced boundary discrimination, and robust inter-class semantic modeling. The main contributions of this work are as follows:

(1) A hierarchical context fusion strategy oriented toward multi-scale objects is proposed, in which complementary context aggregation is introduced at the end of the encoder and within skip connections: ASPP is applied to deep features to enlarge the effective receptive field and strengthen large-scale semantic modeling, while mini-ASPP is embedded into high-resolution skip features for fine-scale context reinforcement and pre-fusion, thereby simultaneously preserving the structural integrity of large objects and the separability of small objects prior to decoding alignment.
(2) To address the tendency of small objects and fine-grained structures in remote sensing scenes to be diluted by down-sampling, MobileViT is integrated into the deep feature extraction process of the encoder, enhancing local texture representation and enabling lightweight global interaction to improve detail modeling, thus yielding more stable recall for small-scale categories such as vehicles.
(3) For scenes characterized by complex class distributions and long-range dependencies, the window-based modeling mechanism of the Swin Transformer is introduced to enhance cross-region semantic consistency under controlled computational cost, thereby improving the modeling capability for large-scale regions and inter-class relationships.

## Related work

### Multi-scale modeling strategies

Remote sensing images often exhibit significant scale variation among objects, with a highly imbalanced distribution ranging from wide-span buildings to small vehicles. This diversity poses a challenge for single-receptive-field-based modeling. To address this, the DeepLab [9] series introduces atrous convolution to construct the ASPP module, enhancing semantic consistency through multi-scale contextual modeling. PSPNet [10] employs a pyramid pooling structure to aggregate global context from the entire image. HRNet [11] maintains high-resolution representations via a parallel multi-branch architecture, thereby improving the detection of small targets. BiSeNetV2 [27] adopts a dual-path strategy, separating the detail and semantic streams to balance edge accuracy and semantic precision. Doğan et al. [28] utilize a multi-branch CNN architecture in urban scenes to improve the recognition of small-scale objects. With the emergence of Transformer-based methods, PVT [29] leverages a hierarchical pyramid structure to enable multi-scale global modeling, while VistaFormer [30] employs a scalable Transformer encoder to handle scale variation in temporal image sequences, further enhancing cross-scale feature representation. The Deep Hybrid Network (DHN) [31] combines the densely connected feature extraction of DenseNet with the encoder–decoder fusion pathway of U-Net to enhance hierarchical representation and detail recovery in high-resolution land-cover segmentation. The feature fusion and context aggregation framework proposed by Khan et al. [32] addresses scale variation through cross-layer feature fusion and employs pyramid pooling to aggregate richer regional context, thereby alleviating misclassification caused by similar farmland textures. Although these methods have achieved notable progress in multi-scale modeling, they still face trade-offs between computational complexity and local semantic accuracy. To mitigate these limitations, we adopt a hybrid strategy by integrating the ASPP module and a compact mini-ASPP module at different depths of the decoder. This enables hierarchical fusion of multi-scale information through deep-level context modeling and shallow-level local feature enhancement.

### Boundary awareness and small object modeling strategies

Blurry boundaries and the tendency of small objects to be lost during downsampling are persistent challenges in remote sensing image segmentation. U-Net [14] and SegNet [15] address this issue by introducing skip connections that pass shallow features to the decoder, thereby preserving edge information. DeepLabv3+ [16] further improves boundary prediction by integrating low-level features into the decoder stage. TransUNet [33] enhances structural awareness by embedding Transformer layers into the U-Net encoder, while UNetFormer [34] combines boundary enhancement modules with multi-scale perception paths to improve contour segmentation accuracy. From an architectural perspective, lightweight networks such as MobileNet [35] and ShuffleNet [36] offer modular and parameter-efficient feature extraction frameworks, making them well-suited for integration and deployment in diverse remote sensing applications with varying resource constraints. A progressive semi-supervised learning method centered on cross-window consistency improves the quality of pseudo-labels and strengthens stable learning of local structures by enforcing consistency constraints among predictions from different windows [37]. An information-fusion-driven perturbation space expansion strategy for remote sensing semantic segmentation guides perturbation augmentation through multi-source information fusion, enabling the model to maintain more robust discriminative capability for fragmented categories under weakly supervised conditions [38]. To enhance the stability and accuracy of boundary representation, we design an edge-guided loss function and embed a mini-ASPP module into the skip connection pathways. This approach strengthens detail preservation and significantly improves the model‘s ability to recognize small targets and delineate boundary regions effectively.

### Global modeling strategies for complex semantic structures

Remote sensing images typically involve a large number of semantic categories with intricate and overlapping spatial distributions. Traditional CNN-based methods are limited in their ability to model long-range dependencies. Vision Transformer (ViT) [39] introduced a pure Transformer-based architecture, demonstrating strong capabilities in capturing long-range semantic relationships; however, it tends to underperform in local detail representation. Swin Transformer [20] addresses this by combining window-based self-attention with a shifted window mechanism, thereby balancing local modeling and cross-region interaction. CF-ViT (Coarse-to-Fine Visual Transformer) [40] retains the CNN backbone while incorporating Transformer-style inductive bias to improve semantic consistency. SCTNet [41] introduces a lightweight semantic enhancement branch to boost the discriminative power of backbone features. RSFormer [42] enhances semantic decoupling by integrating multi-scale fusion with positional attention. RSAM-Seg [43] leverages prior knowledge to model high-frequency features, while VistaFormer [30] adopts a position-free encoding strategy to reduce reliance on spatial structure priors. These methods have collectively advanced the understanding of complex class structures, spatial context, and long-range semantic relationships in remote sensing imagery. DNAS [44] decouples the neural architecture search process to obtain effective network structures at a reduced search cost, and its feasibility has been validated on high-resolution remote sensing semantic segmentation tasks. For multi-source remote sensing data, Tuple Perturbation-Based Contrastive Learning [45] enhances cross-modal representational consistency through the combination of tuple perturbation and contrastive learning, thereby improving robustness under complex semantic conditions. In this work, we incorporate Swin Transformer [20] into the upper layers of the encoder to further model the output features of MobileViT [17], constructing a hierarchical semantic extraction path that progresses from local to global representations. This design not only preserves architectural compactness but also enhances the model‘s capacity for inter-class relational modeling and contextual consistency.

## Method

### Overview of the network architecture

**Fig 1:**
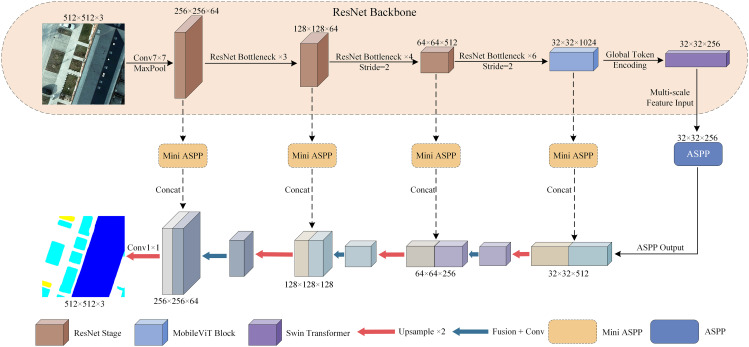
Schematic Diagram of the RTAS-Net Architecture.

This study proposes a deep neural network architecture for semantic segmentation of remote sensing images, termed RTAS-Net (ResNet-Transformer-ASPP Segmentation Network). The model adopts a typical encoder–decoder structure to jointly enable multi-scale feature extraction, contextual modeling, and precise boundary segmentation. As illustrated in Fig.1, the encoder employs ResNet-50 as the backbone and extracts high-level semantic features through a series of residual blocks. At the higher stages of feature extraction, the network incorporates two Transformer-based modules: the MobileViT block and the Swin Transformer. The former enhances the model‘s capacity to capture local structural details, while the latter employs a window-based self-attention mechanism to model global dependencies across the image. This dual-transformer design improves the representation of inter-object semantic relationships in complex remote sensing scenes.

To further enrich the multi-scale representation of features, an Atrous Spatial Pyramid Pooling (ASPP) module is introduced at the end of the encoder. It leverages parallel dilated convolutions with varying receptive fields to capture global contextual information, which is subsequently fused with features from different semantic levels. Simultaneously, an enhanced skip-connection mechanism is adopted, whereby features from different encoder stages are passed to the decoder via Mini-ASPP modules. This facilitates effective alignment between shallow spatial details and deep semantic features. In the decoder, the network performs a series of upsampling, feature fusion, and convolution operations to progressively restore spatial resolution and generate pixel-level predictions. The final output is produced by a 1×1 convolution layer that generates a semantic segmentation map with the same spatial resolution as the input image. During training, a composite loss function is employed, which jointly accounts for class imbalance, boundary ambiguity, and prediction confidence. This enhances the model‘s discrimination capability in edge regions and improves overall segmentation accuracy.

Importantly, RTAS-Net is not a simple aggregation of individual functional modules. Instead, it establishes a complementary and collaborative mechanism across the encoder, skip connections, and context modeling paths by leveraging the strengths of each module. MobileViT focuses on shallow-level local structure representation, thereby enhancing edge and detail modeling. Swin Transformer constructs regional semantic associations through window-based attention, improving global consistency. ASPP and Mini-ASPP reinforce semantic features at different depths, enabling multi-scale enhancement across both the backbone and skip pathways. The network structure of RTAS-Net is shown in Fig 1.

### ResNet50-based improved encoder

To enhance feature representation and improve the generalization performance in remote sensing semantic segmentation tasks, we optimize the encoder component of the classic U-Net architecture. The proposed encoder adopts ResNet-50 as the backbone and integrates both MobileViT blocks and Swin Transformer modules at the higher semantic levels. The MobileViT block is used to strengthen local structure modeling and boundary detail extraction, while the Swin Transformer leverages window-based attention to model long-range semantic dependencies across classes. This hybrid encoding design balances local detail preservation and global context understanding, significantly improving segmentation accuracy and semantic consistency in complex remote sensing scenes, all while maintaining manageable structural complexity.

The encoder is specifically designed as follows: ResNet-50 is selected as the backbone to replace the plain convolutional stacking used in the original U-Net encoder. Conventional convolutional architectures often fall short when applied to complex remote sensing imagery—such as high-resolution representations of urban areas, farmlands, or water bodies—due to limited receptive fields and insufficient feature representation capacity. In contrast, ResNet-50, with its deep residual architecture, offers enhanced feature extraction while maintaining stable gradients and efficient training. It is thus better suited to capture the rich semantic content, spatial variation, and intricate textures characteristic of remote sensing images. In our implementation, the first three stages of ResNet-50 are preserved, including the initial convolutional layer (conv1) and subsequent layers (layer1 through layer3). These layers, composed of deep residual units, enhance the encoder‘s ability to perceive local spatial structures. Their outputs also serve as inputs for skip connections, providing the decoder with multi-scale edge details and intermediate semantic features. The feature extraction process at this stage can be mathematically expressed please see (1):


$$\begin{array}{cc} F_{1} & =ReLU\left(BN\left({Conv}_{7\times 7,s=2}(x)\right)\right)\\ F_{2} & =ℬ\left(MaxPool(F_{1})\right)\\ F_{3} & =ℬ(F_{2}),\;F_{4}=\beta (F_{3})\\ ℬ & =BN\left({Conv}_{3}\left(ReLU\left(BN\left({Conv}_{2}\left(ReLU\left(BN\left({Conv}_{1}(F_{2})\right)\right)\right)\right)\right)\right)\right) \end{array}$$ (1)

Here, the input image is denoted as $x\in {\mathbb{R}}^{3\times H\times W}$, and ${Conv}_{7\times 7,\;s=2}(\cdot )$, BN(·), ReLU(·) respectively represent a 7×7 convolution with stride 2, batch normalization, and an activation operation, while MaxPool(·) denotes max pooling.B(·) denotes the stacked Bottleneck residual blocks of ResNet50 (corresponding to one stage), and $F_{1}$, $F_{2}$, $F_{3}$ represent the feature maps extracted from layer1, layer2, and layer3 of the ResNet backbone, respectively, denoted as $F_{s}\in {\mathbb{R}}^{C_{s}\times H_{s}\times W_{s}}$. Under the input setting of this study (512×512), the spatial resolution of the layer3 output is approximately $H_{3}=W_{3}=32$ with a channel dimension of $C_{3}=1024$, and this feature map serves as the input basis for the subsequent MobileViT and Swin modules.

To further enhance the model’s ability to capture global contextual information, the original fourth stage of ResNet (i.e., layer4, composed of multiple Bottleneck modules) is replaced with a MobileViT Block in the higher-level stages of the encoder. Although the traditional Bottleneck structure provides strong semantic abstraction capabilities, it is inherently limited by its reliance on local receptive fields, making it insufficient for capturing long-range dependencies and globally distributed features in complex remote sensing imagery. The MobileViT module combines the strengths of Convolutional Neural Networks (CNNs) and Transformers. While preserving the spatial structural awareness offered by CNNs, it introduces cross-region self-attention mechanisms that significantly enhance the global consistency and semantic completeness of the feature representations. This module offers the following advantages: (1) It enables efficient fusion of local and global information while maintaining spatial resolution; (2) Compared to pure Transformer-based designs, it provides better structural adaptability and parameter efficiency, facilitating integration into existing architectures; (3) It is highly compatible with ResNet, allowing seamless incorporation into the original backbone. This conversion stage can be expressed mathematically as in equation (2):


$$\begin{array}{cc} F_{5} & =ℳ(F_{4})\\ ℳ & =\left\{ \begin{array}{c} F_{\text{local}}={\text{Conv}}_{3\times 3}(x),\;F_{\text{proj}}={\text{Conv}}_{1\times 1}(F_{\text{local}})\in {\mathbb{R}}^{d\times H\times W}\\ T=\text{Unfold}(F_{\text{proj}})\in {\mathbb{R}}^{N\times P\times d},\;\hat{T}=\text{TransformerEncoder}(T)\\ {\hat{F}}_{\text{global}}=\text{Fold}(\hat{T})\in {\mathbb{R}}^{d\times H\times W},\;F_{\text{fusion}}=\text{Concat}(F_{\text{local}},{\hat{F}}_{\text{global}})\\ Y={\text{Conv}}_{1\times 1}(F_{\text{fusion}})\in {\mathbb{R}}^{C\times H\times W} \end{array}\right. \end{array}$$ (2)

Here, ℳ(·) denotes a MobileViT block. Let the input feature be $F_{4}\in {\mathbb{R}}^{C\times H\times W}$ (in this work, C=1024, and typically H=W=32 for a 512×512 input). The input is first processed by a local convolution to obtain $F_{\text{local}}\in {\mathbb{R}}^{C\times H\times W}$, which is then projected to the Transformer embedding dimension *d* via a 1×1 convolution to yield $F_{\text{proj}}\in {\mathbb{R}}^{d\times H\times W}$. Subsequently, $F_{\text{proj}}$ is partitioned into non-overlapping p×p patches and unfolded to form a token sequence $T\in {\mathbb{R}}^{N\times P\times d}$, where $P=p^{2}$ denotes the number of tokens within each patch and N=(H/p)·(W/p) denotes the number of patches. The Transformer encoder models *T* to produce $\hat{T}\in {\mathbb{R}}^{N\times P\times d}$, which is then folded back to the spatial feature map ${\hat{F}}_{\text{global}}\in {\mathbb{R}}^{d\times H\times W}$. Finally, ${\hat{F}}_{\text{global}}$ is concatenated with $F_{\text{local}}$ along the channel dimension and fused to output the enhanced feature representation of MobileViT.

Through the integration of MobileViT, the encoder enhances the representation capacity of deep semantic features without significantly increasing model complexity. This substitution improves the model‘s perceptual robustness, particularly when dealing with low-contrast targets, large-scale ground objects, and regions with blurred boundaries.

Building upon the preliminary global awareness provided by the MobileViT block, we further introduce the Swin Transformer module to refine its output features. The Swin Transformer employs a window-based self-attention mechanism with a shifted window strategy, enabling cross-region feature interaction while maintaining computational efficiency. This design allows the network to capture broader contextual dependencies, which are crucial in remote sensing imagery where object categories are diverse, scale variations are significant, and spatial pixel distributions are highly non-uniform. Conventional convolution operations often struggle to model long-range semantic relationships between distant pixels under such conditions. In this context, the Swin Transformer demonstrates unique advantages: on one hand, its localized window attention facilitates focused modeling of structural information; on the other, the shifted window mechanism enables cross-region fusion, enhancing the consistency of recognition for spatially continuous objects such as roads and buildings. Compared to MobileViT—which emphasizes local structure modeling—the Swin Transformer focuses more on building global semantic dependencies. The two modules thus complement each other within the architecture, jointly improving the expressiveness of high-level feature representations.

To ensure seamless integration, the output features from the MobileViT block are first passed through a 1×1 convolution layer to align channel dimensions as shown in Equation (3):


$${\tilde{F}}_{5}=\text{ReLU}(\text{BN}({\text{Conv}}_{1\times 1}(F_{5})))$$ (3)

Then, the features are fed into the Swin Transformer encoder for global context modeling, and residual connections are applied to form the final semantic featuresas shown in Equation (4):


$$\begin{array}{cc} F_{6} & =𝒮({\tilde{F}}_{5})+{\tilde{F}}_{5}\\ 𝒮 & =\left\{ \begin{array}{c} x_{1}=x+W-\text{MSA}(\text{LN}(x))\\ Y=x_{1}+\text{FFN}(\text{LN}(x_{1})) \end{array}\right. \end{array}$$ (4)

Here, ${Conv}_{1\times 1}(\cdot )$ is used for channel alignment, mapping the MobileViT output feature $F_{5}\in {\mathbb{R}}^{C\times H\times W}$ to the Transformer dimension *d* to obtain ${\tilde{F}}_{5}\in {\mathbb{R}}^{d\times H\times W}$ (in this work, d=256 and H=W=32). 𝒮(·) denotes a Swin Transformer block, which first rearranges the feature into ${\mathbb{R}}^{H\times W\times d}$, then partitions it into windows of size M×M, applies window-based multi-head self-attention (W-MSA) within each window, and realizes cross-window information interaction through the shifted window strategy (SW-MSA), finally restoring it to ${\mathbb{R}}^{d\times H\times W}$. In the formulation, the residual connection $F_{6}=𝒮({\tilde{F}}_{5})+{\tilde{F}}_{5}$ is used to stabilize training and preserve local inductive bias.

This component, functioning as the Global Token Encoding stage of the network, substantially enhances the encoder‘s capacity to model long-range semantic dependencies and global contextual information. It provides structural priors and semantic guidance for subsequent multi-scale feature fusion.

**Fig 2:**
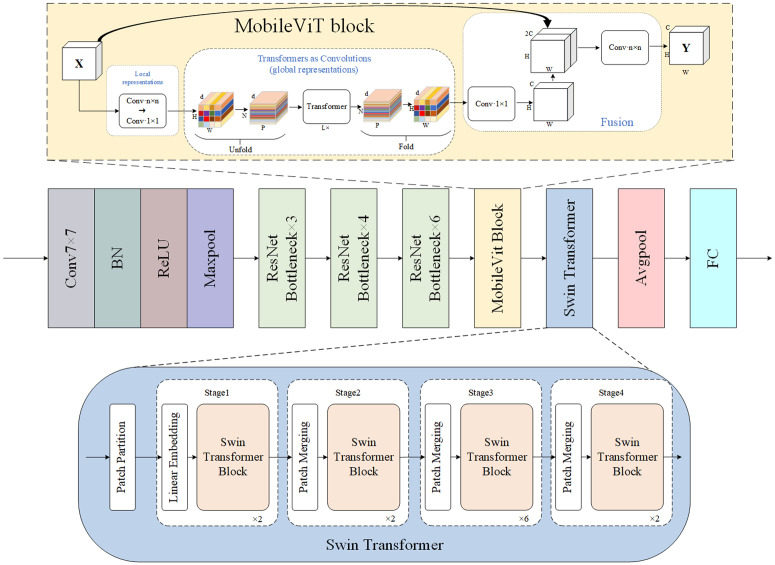
Schematic Diagram of the Improved Encoder Structure and Its Components.

The encoder is designed to function complementarily and collaboratively with the decoder, achieving both functional synergy and path-level coordination. On one hand, the combined use of MobileViT and Swin Transformer modules enhances the semantic abstraction and global modeling capabilities of deep features, improving the representation of complex object structures and long-range dependencies in remote sensing imagery. On the other hand, shallow-layer outputs from ResNet preserve high-resolution edge details and texture features through skip connections, providing crucial spatial structural cues to support accurate reconstruction during decoding. Given the complexity of object boundaries and the significant scale variations in remote sensing scenes, relying solely on high-level semantic features may lead to boundary ambiguity. To mitigate this, a compact mini-ASPP module is incorporated into the decoder to strengthen the multi-scale representation of shallow features. By guiding information flow from varying receptive fields through the skip connections into the decoding process, this module enhances the network‘s ability to detect small objects and delineate complex boundaries. It effectively improves contextual integration along the decoding path while maintaining the precision of feature representation, ultimately leading to better segmentation performance. An illustration of the improved encoder architecture and its constituent modules is provided in Fig 2.

### ASPP and mini-ASPP decoder module structure

To enhance the representational capacity of deep semantic features and improve the model‘s adaptability to objects of varying scales, this study introduces an Atrous Spatial Pyramid Pooling (ASPP) module at the highest semantic level of the encoder output. Additionally, mini-ASPP modules are embedded within each skip connection of the decoder, enabling joint modeling of global and local scale information.

The ASPP module is positioned at the end of the encoder, specifically applied to the feature output from the Swin Transformer, which contains the most abstract semantic representations. By configuring multiple parallel convolutional branches with different dilation rates, ASPP effectively expands the receptive field and introduces multi-scale contextual information without sacrificing spatial resolution. The mathematical formulation of each dilated convolution branch as shown in Equation (5):


$$y[i]=\sum_{\tilde{k}}x[i+r\cdot \tilde{k}]\cdot w[\tilde{k}]$$ (5)

Here, *x* denotes the input feature map; *y* represents the output feature map; *w* is the convolution kernel weight; *r* is the dilation rate; $\tilde{k}$ indicates the kernel index; and *i* denotes the input index.

The combination of the output features from the ASPP module as shown in Equation (6):


$$F_{\text{ASPP}}=\text{Concat}\left(f_{1\times 1},f_{3\times 3}^{\left(\gamma_{1}\right)},f_{3\times 3}^{\left(\gamma_{2}\right)},f_{3\times 3}^{\left(\gamma_{3}\right)},f_{\text{GAP}}\right)$$ (6)

Here, $f_{1\times 1}$ represents the 1 × 1 convolution branch; $f_{3\times 3}^{\left(\gamma_{i}\right)}$ denote the 3 × 3 convolution branches with dilation rates of 6, 12, and 18, respectively; $f_{\text{GAP}}$ indicates the feature from the global average pooling branch; and Concat represents concatenation along the channel dimension.

**Fig 3:**
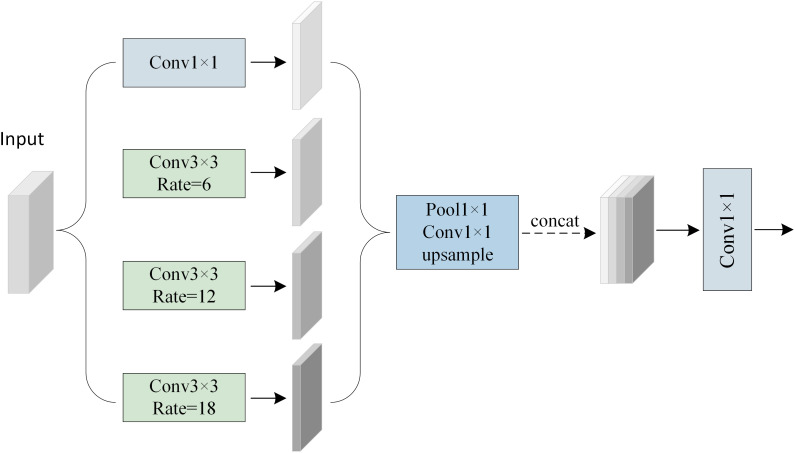
The Structure of the ASPP Module.

This design significantly enhances the model’s capacity to perceive targets exhibiting substantial scale variations in remote sensing imagery, particularly improving segmentation continuity and accuracy in regions characterized by blurred boundaries and densely distributed small objects. As illustrated in Fig 3, the ASPP module comprises multiple parallel branches, including a 1 × 1 convolution, several 3 × 3 convolutions with varying dilation rates, and a global average pooling operation. These feature branches are subsequently fused to produce multi-scale perceptual representations.

**Fig 4:**
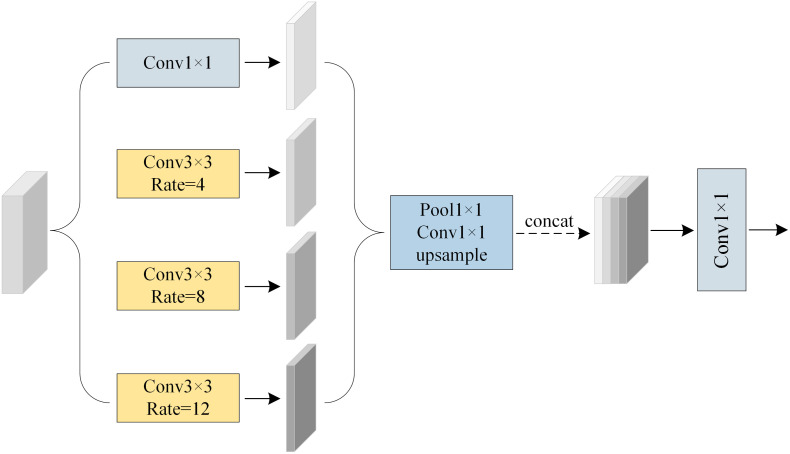
The Structure of the mini-ASPP Module.

During the decoding phase, to mitigate the semantic gap between shallow encoder features and the upsampled decoder features—and to avoid information confusion caused by direct concatenation— mini-ASPP module is introduced at each skip connection. This module inherits the multi-scale modeling concept of the original ASPP while adopting smaller dilation rates and a reduced number of channels to improve adaptability. Its primary goal is to enhance the contextual awareness of shallow features, making them more suitable for alignment and fusion with high-level semantic features. By guiding multi-scale semantic information into the reconstruction of skip connection features, the mini-ASPP module effectively improves the discriminative power of the fused regions. It demonstrates particularly strong performance in boundary detail restoration and small-object perception, thereby enhancing the local modeling capability and segmentation accuracy of the decoder. The architecture of the mini-ASPP module is illustrated in Fig 4.

### Composite loss function

In remote sensing semantic segmentation tasks, models often encounter challenges such as imbalanced class distributions, varying object scales, and blurred boundaries—all of which can adversely impact segmentation accuracy and generalization performance. To address these issues in complex scenarios, this study introduces a composite loss function that integrates Focal Loss, Dice Loss, and edge-guided Binary Cross-Entropy (BCE) Loss. This combined formulation jointly supervises the training process from three key perspectives: classification precision, region-level consistency, and boundary sensitivity.

Focal Loss is primarily employed to mitigate the effects of class imbalance. Compared to standard cross-entropy loss, it introduces a modulating factor that down-weights the contribution of easily classified samples, thereby directing the model‘s focus toward hard examples. The formulation as shown in Equation (7):


$${ℒ}_{\text{focal}}=-\alpha_{t}(1-p_{t}{)}^{\gamma }\log(p_{t})$$ (7)

Here, $p_{t}$ denotes the predicted probability, $\alpha_{t}$ controls the inter-class weighting, and *γ* is the focusing parameter, typically set to 2. This loss function reduces the weight of easily classified samples while enhancing the model‘s learning effectiveness on small targets and boundary regions.

Dice Loss emphasizes the overlap between the predicted region and the ground truth, effectively improving segmentation performance for small objects and irregular regions. It is defined as shown in Equation (8):


$${ℒ}_{\text{dice}}=1-\frac{2\sum_{i}p_{i}g_{i}+\epsilon }{\sum_{i}p_{i}+\sum_{i}g_{i}+\epsilon }$$ (8)

Here, $p_{i}$ and $g_{i}$ represent the pixel-level probability distributions of the prediction and ground truth, respectively, and *ϵ* is a smoothing term. Dice Loss is particularly well-suited for optimizing the segmentation accuracy of small-area targets, compensating for the limitations of Focal Loss in preserving structural integrity.

In addition, considering the widespread presence of blurred boundaries and object overlap in remote sensing images, an edge supervision mechanism based on the Sobel operator is designed. Specifically, pseudo-edge maps are generated from both the prediction results and the ground truth labels, and the binary cross-entropy loss is computed as shown in Equation (9):


$${ℒ}_{\text{edge}}=\text{BCE}\left(\text{Sobel}(\hat{Y}),\text{Sobel}(Y)\right)$$ (9)

Here, $\hat{Y}$ denotes the model output, and *Y* represents the ground truth labels.

Edges are extracted using the Sobel operator and incorporated into the BCE loss calculation. This loss guides the model to focus on structural contours without introducing additional annotation cost, effectively improving the quality of boundary predictions.

Finally, the three loss components are combined in a weighted manner to form the total loss function, as shown in Equation (10):


$${ℒ}_{\text{total}}={ℒ}_{\text{focal}}+\lambda_{1}{ℒ}_{\text{dice}}+\lambda_{2}{ℒ}_{\text{edge}}$$ (10)

Here, $\lambda_{1}$ and $\lambda_{2}$ are the weighting coefficients for the Dice Loss and edge loss, respectively, and can be adjusted based on the characteristics of the dataset.

Experimental results demonstrate that this composite loss strategy offers better convergence and boundary sensitivity compared to single-loss approaches, with particularly notable improvements in small object recognition and edge refinement tasks.

## Experiments and results

### Datasets

To comprehensively evaluate the segmentation accuracy and the effectiveness of the structural design of RTAS-Net in remote sensing semantic segmentation tasks, experiments are conducted on three public datasets. ISPRS Potsdam and ISPRS Vaihingen, as high-resolution aerial imagery benchmarks, cover typical urban land-cover categories and complex boundary scenarios, and are used for main performance comparisons and ablation studies. The LoveDA dataset is further selected as a cross-dataset validation platform, whose more pronounced scene discrepancies and data style variations are used to examine the stability of the model under different data distributions and the robustness of the training configuration.

### ISPRS potsdam dataset

The Potsdam dataset, provided by ISPRS in Germany, consists of 38 true orthophoto (TOP) images, each with a resolution of 6000 × 6000 pixels and a spatial resolution of 5 cm per pixel. The dataset covers representative urban elements such as buildings, roads, vegetation, and water bodies, and includes four spectral bands: red (R), green (G), blue (B), and near-infrared (NIR). It also provides auxiliary Digital Surface Model (DSM) data to support multimodal feature extraction and analysis. The ground truth annotations include six semantic classes: buildings (blue), trees (green), low vegetation (cyan), impervious surfaces (white), cars (yellow), and background (red).

### ISPRS vaihingen dataset

The Vaihingen dataset, also released by ISPRS, contains 33 high-resolution aerial images, each with approximate dimensions of 2000 × 2500 pixels and a spatial resolution of 9 cm per pixel. Compared to Potsdam, the Vaihingen dataset predominantly features residential areas and medium-to-low density urban structures. It is characterized by more complex object boundaries and closer inter-class spatial distributions, making it particularly suitable for evaluating model generalization under structural complexity and occlusion. The category definitions remain consistent with those in the Potsdam dataset, supporting strong cross-dataset transferability. In this study, the Vaihingen dataset is also used for transfer evaluation under varied imaging and resolution conditions.

### LoveDA dataset

LoveDA is a dataset released by the RSIDEA team of Wuhan University (NeurIPS 2021 Datasets and Benchmarks). It contains a total of 5,987 high–spatial-resolution optical remote sensing images, each with a size of 1024×1024 pixels and a ground sampling distance of approximately 0.3 m/pixel. The dataset is constructed based on historical Google Earth imagery and covers 18 urban and rural administrative regions across three cities—Nanjing, Changzhou, and Wuhan—with a total area of about 536.15 km². It includes two domains, Urban and Rural, and is suitable for cross-domain generalization studies such as land-cover semantic segmentation and unsupervised domain adaptation. The imagery provides three spectral bands: red (R), green (G), and blue (B). Under the official benchmark setting, the annotations include semantic labels for seven categories: background, building, road, water, barren, forest, and agriculture.

### Experimental setup

To verify the effectiveness and robustness of the proposed RTAS-Net in remote sensing semantic segmentation tasks, experiments are conducted on three public datasets: ISPRS Potsdam, ISPRS Vaihingen, and LoveDA. A unified experimental setting is adopted, and the experimental procedure is described in terms of data preprocessing and dataset partitioning, as well as training configuration and evaluation metrics, to ensure the comparability of results across different datasets.

### Data preprocessing and dataset partitioning

Due to the ultra-large size of remote sensing images, directly inputting them into deep neural networks imposes a heavy burden on GPU memory and limits training efficiency and stability. Therefore, this study adopts a sliding window cropping strategy to process the original images, converting large-scale images into smaller patches suitable for model training. To preserve contextual continuity and alleviate edge information loss, overlapping regions are introduced during cropping. The specific preprocessing steps are as follows:

Potsdam Dataset: A total of 38 original large-scale images with a resolution of 6000 × 6000 pixels were processed. Using an overlap ratio of 0.7, each image was cropped into patches of 512 × 512 pixels, resulting in 7448 image blocks. These were then organized in VOC format (including JPEGImages, SegmentationClass, and ImageSets directories) and divided into 5958 training samples and 1490 validation samples.

Vaihingen Dataset: This dataset contains 33 images, which were cropped using the same method as the Potsdam dataset. A total of 4693 image blocks were generated, with 3754 used for training and 939 for validation. This preprocessing strategy ensures consistency between the two datasets in terms of image preparation and training structure, laying a foundation for fair and comparable analysis.

LoveDA dataset: To maintain consistency with the aforementioned experiments in terms of input format, a sliding-window cropping strategy is also adopted to generate fixed-size image patches, and the dataset split is constructed following the same training pipeline. Ultimately, 3,352 image patches are used for training and 839 for validation.

### Training settings and evaluation metrics

During model training, the input image size is uniformly set to 512 × 512, with a batch size of 8. The Adam optimizer is used with an initial learning rate of 1e-4, dynamically adjusted using a cosine annealing learning rate scheduler. The number of training epochs is set to 100. All experiments are conducted on a machine equipped with an NVIDIA GeForce RTX 4070 Ti SUPER GPU with 16GB of memory.

To ensure fairness and consistency in comparison, all models are trained under the same settings and evaluated using a unified validation set. This study adopts the following four widely used semantic segmentation metrics for quantitative performance analysis:

Mean Intersection over Union (mIoU): Intersection over Union (IoU) is a widely used metric to evaluate the overlap between predicted segmentation results and ground truth labels for each class. It is defined as:


$${IoU}_{i}=\frac{TP_{i}}{TP_{i}+FP_{i}+FN_{i}}$$ (11)

The mean Intersection over Union (mIoU) is calculated as the average IoU across all classes:


$$mIoU=\frac{1}{C}\sum_{i=1}^{C}{IoU}_{i}$$ (12)

Here, TP, FP, and FN denote true positives, false positives, and false negatives, respectively. Specifically, TP refers to the number of pixels correctly predicted as belonging to a given class; FP refers to the number of pixels incorrectly predicted as belonging to that class; and FN refers to the number of pixels that belong to the class in the ground truth but were not predicted as such. C represents the total number of classes. This metric quantifies the degree of overlap between the predicted segmentation and the ground truth, and is regarded as one of the most representative indicators of segmentation accuracy.

Mean F1-Score (mF1): The F1 score measures the balance between the model‘s precision and recall:


$${Precision}_{i}=\frac{TP_{i}}{TP_{i}+FP_{i}},\;{Recall}_{i}=\frac{TP_{i}}{TP_{i}+FN_{i}}$$ (13)

$$F1_{i}=\frac{2\cdot {Precision}_{i}\cdot {Recall}_{i}}{{Precision}_{i}+{Recall}_{i}}=\frac{2\cdot TP_{i}}{2\cdot TP_{i}+FP_{i}+FN_{i}}$$ (14)

$$mF1=\frac{1}{C}\sum_{i=1}^{C}F1_{i}$$ (15)

The mean F1 score emphasizes the model‘s recall and classification accuracy for each individual class. It serves as a strong complement to the IoU metric, particularly offering advantages in evaluating the delineation of object boundary details. All metrics are computed at the pixel level and averaged across all categories to provide a comprehensive and fair performance evaluation.

Overall Accuracy (OA): Overall Accuracy measures the proportion of correctly predicted pixels over the total number of predictions and is defined as:


$$OA=\frac{\sum_{i=1}^{C}TP_{i}}{\sum_{i=1}^{C}(TP_{i}+FP_{i}+FN_{i}-TP_{i})}=\frac{\sum_{i=1}^{C}TP_{i}}{\sum_{i=1}^{C}N_{i}}$$ (16)

OA serves as a global metric that reflects the model‘s overall classification accuracy across all pixels. It is particularly useful for evaluating the consistency of full-scene predictions.

Model Parameters (Params): This metric quantifies the total number of trainable parameters within a model and is commonly used to assess model complexity and computational resource requirements. Each parameter corresponds to a learnable weight updated during training. A larger number of parameters typically implies greater representational capacity, but also leads to higher demands on computational resources. The total number of parameters is calculated as:


$$Params=\sum_{l=1}^{L}({Weights}_{l}+{Biases}_{l})$$ (17)

Where L denotes the number of network layers. Parameters from common layers such as convolutional layers, fully connected layers, and attention mechanisms are all included in the count. In this work, the Params metric is used alongside mIoU, mF1, and OA to further evaluate the architectural efficiency and deployment feasibility of the proposed model.

All metrics are computed at the pixel level and averaged across all categories to provide a comprehensive and fair performance evaluation.

### Comparative experiments

To more comprehensively evaluate the segmentation capability of RTAS-Net on the two standard datasets ISPRS Potsdam and Vaihingen, this study selects comparative methods that cover different representative design paradigms. PSPNet [10], DeepLabv3+ [16], and HRNet [11], as classical convolutional network baselines, reflect the fundamental performance of multi-scale context modeling and high-resolution feature extraction. BANet [46], ABCNet [47], SAINet [48], and CVMH-Unet [49] focus more on boundary refinement, feature interaction, and class discrimination in remote sensing scenes, which are directly comparable to the fine-grained structural representation and regional consistency issues addressed in this work. MAResU-Net [50] and UNetFormer [34] represent more recent U-Net variants, embodying strategies of multi-scale reorganization and Transformer-based long-range dependency modeling, respectively, and are more closely aligned with our design that introduces MobileViT and Swin Transformer in the encoder together with multi-scale context modules. Based on these selections, the comparison set includes both general baseline models and recent methods with key components and modeling objectives similar to those of this study, enabling a more objective assessment of the performance gains and associated costs of RTAS-Net under a unified experimental setting. In addition, cross-dataset validation and robustness analysis of training configurations are conducted on the LoveDA dataset to examine the stability of the model under more complex scenarios and pronounced style variations.

### Comparative experiments on the potsdam dataset

**Fig 5:**
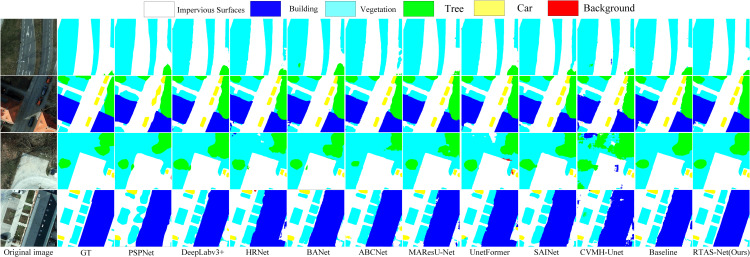
Qualitative comparison results of different methods on the Potsdam dataset.

Table 1 reports the results of all methods on the ISPRS Potsdam dataset. RTAS-Net achieves the best performance in terms of mIoU, mF1, and OA, with values of 87.14%, 92.98%, and 92.63%, respectively. From a class-wise perspective, the improvements are mainly concentrated on objects with complex boundaries and pronounced scale variation, such as vehicles and trees, whose IoU scores are higher, typically corresponding to fewer missed detections and misclassifications. The visualization results in Fig 5 are consistent with this observation: in areas with dense vehicles and at building–road interfaces, RTAS-Net produces more complete contours and more continuous edges. In regions where vegetation and impervious surfaces are interlaced, the class transitions are cleaner and local “adhesion” between categories is reduced. Since the overall accuracy on the Potsdam dataset is already relatively high, improvements in average metrics are often manifested as reductions of errors in challenging regions, which aligns with the improvement priorities in remote sensing mapping scenarios. The corresponding computational cost is provided in the subsequent efficiency analysis, facilitating trade-off decisions according to application requirements.

**Table 1:** Quantitative comparison on the ISPRS Potsdam dataset. The accuracy for each class is presented in the form of IoU (%). The best results are highlighted in bold.

Method	Impervious surface	Building	Low vegetation	Tree	Car	mIoU	mF1	OA
PSPNet [10]	70.68	71.11	73.48	90.48	82.86	77.17	86.94	88.81
DeepLabv3+ [16]	81.89	71.45	73.96	90.95	83.84	78.24	87.57	89.16
HRNet [11]	80.43	70.78	73.29	88.88	82.37	70.44	80.58	90.22
BANet [46]	82.15	73.33	75.80	91.96	85.37	80.79	89.21	87.26
ABCNet [47]	79.97	68.67	70.09	87.19	82.26	74.58	85.10	87.97
UnetFormer [34]	80.77	69.26	72.82	89.80	82.74	73.40	83.78	87.87
SAINet [48]	77.98	69.54	72.75	89.93	82.54	75.85	88.22	85.97
CVMH-Unet [49]	80.11	70.14	70.28	90.13	85.44	74.85	82.66	85.56
MAResU-Net [50]	80.17	70.55	73.37	89.96	82.54	72.58	82.93	87.97
Baseline	84.49	76.25	78.49	93.36	87.95	81.28	89.45	91.33
**RTAS-Net (Ours)**	**86.07**	**77.11**	**80.15**	**94.95**	**88.55**	**87.14**	**92.98**	**92.63**

### Comparative experiments on the vaihingen dataset

**Fig 6:**
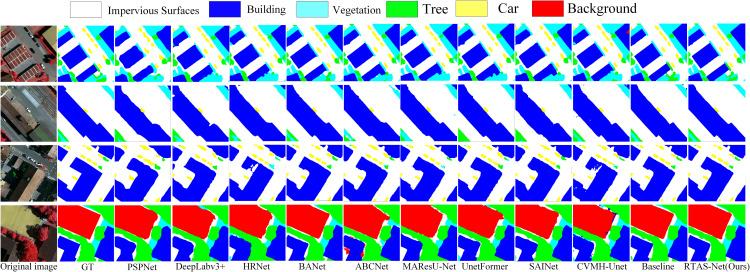
Qualitative comparison results of different methods on the Vaihingen dataset.

On the ISPRS Vaihingen dataset (Table 2), RTAS-Net also achieves the best performance, with an mIoU of 92.09%, an mF1 of 95.85%, and an OA of 96.31%. Compared with multiple competing methods, its advantages are more prominently reflected in structural continuity and boundary quality. As illustrated in Fig 6, RTAS-Net exhibits greater stability at building edges, linear road structures, and the interfaces between vegetation and background, with fewer instances of local fragmentation and boundary drift. In regions with strong interference, such as areas where rooftops are adjacent to tree canopies or where occlusion is prevalent, the predicted regions are closer to the true contours. Changes in overall quantitative metrics are not fully equivalent to variations in visual quality, and the improvements on the Vaihingen dataset are mainly manifested as enhanced robustness to complex details and boundary regions.

**Table 2:** Quantitative comparison on the ISPRS Vaihingen dataset. The accuracy for each class is presented in the form of IoU (%). The best results are highlighted in bold.

Method	Impervious surface	Building	Low vegetation	Tree	Car	mIoU	mF1	OA
PSPNet [10]	57.03	80.48	76.06	91.42	84.99	79.20	87.94	90.79
DeepLabv3+ [16]	73.04	82.68	78.43	92.69	88.20	83.62	90.94	92.29
HRNet [11]	72.71	78.98	72.22	90.85	85.65	80.63	89.12	90.23
BANet [46]	83.13	88.58	86.59	95.49	92.39	89.58	94.45	95.24
ABCNet [47]	74.32	81.57	75.52	92.48	87.04	81.96	89.95	91.50
UnetFormer [34]	76.24	82.80	78.25	93.11	88.42	84.23	91.33	92.40
SAINet [48]	67.36	82.39	77.92	92.12	87.21	81.97	89.88	91.88
CVMH-Unet [49]	79.68	79.75	73.27	91.91	86.52	80.17	88.81	90.77
MAResU-Net [50]	75.74	81.56	76.70	92.91	87.79	83.49	90.88	91.88
Baseline	85.49	90.06	88.43	96.30	93.44	91.14	95.33	95.94
**RTAS-Net (Ours)**	**87.26**	**90.79**	**89.51**	**96.63**	**94.01**	**92.09**	**95.85**	**96.31**

### Ablation study

To demonstrate that the performance gains of RTAS-Net arise from “clearly delineated cooperative module functions” rather than simple component stacking, this study conducts ablation experiments along two main lines. First, modules are progressively added from Models A to E to analyze which types of modules contribute to the observed performance improvements. Second, without adding or removing modules, the placement and connection order of MobileViT and Swin Transformer are adjusted to examine how the sequence in which features pass through these modules affects performance. Visualization and efficiency statistics are then incorporated to report not only accuracy results but also the sources of improvement, together with the associated computational and resource costs. In addition, the U-Net baseline refers to an implementation based on the standard U-Net encoder–decoder framework, in which ResNet50 is used at the encoder to extract multi-scale features and the decoder adopts the same up-sampling and skip-connection fusion structure.

### Ablation study on the potsdam dataset

Table 3 presents the performance of different module combinations on the Potsdam dataset, evaluated using four metrics: mIoU, mF1, OA, and Params.

**Table 3:** Performance Comparison of Different Module Combinations (Potsdam).

Model	Structural description	mIoU (%)	mF1 (%)	OA (%)	Params (M)
A	U-Net baseline	81.28	89.45	91.33	43.93
B	A + MobileViT	84.57	91.54	92.06	51.66
C	B + Swin Transformer	84.92	91.74	92.22	53.89
D	C + ASPP	85.64	92.15	92.25	69.96
E	D + mini-ASPP (ours)	**87.14**	**92.98**	**92.63**	**100.85**

**Fig 7:**
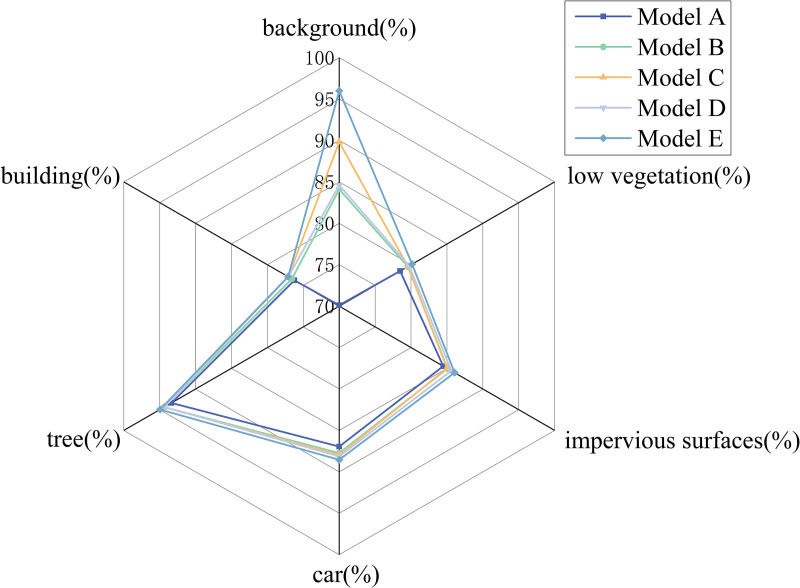
Radar Chart of Per-Class IoU for Ablation Models on the Potsdam Dataset.

To further illustrate the differences in performance across fine-grained semantic categories for each module combination, a radar chart is presented in Fig 7. The chart displays the IoU scores for six classes—background, vehicles, trees, low vegetation, buildings, and impervious surfaces—comparing models from model A (U-Net baseline) to model E (complete RTAS-Net).

Fig 7 illustrates the IoU distribution across six semantic categories on the Potsdam dataset for different module configurations, offering a fine-grained analysis of class-level performance variations following the integration of each architectural component. The results reveal that incorporating the MobileViT block significantly improves the model‘s ability to detect small objects such as vehicles. The Swin Transformer enhances semantic consistency for large-area categories like impervious surfaces and low vegetation. The ASPP module facilitates multi-scale contextual fusion, particularly benefiting segmentation around structurally complex boundaries. The addition of the mini-ASPP module further strengthens shallow feature enhancement within skip connections, yielding notable improvements in texture-rich regions such as buildings and trees. Overall, the progressive performance gains observed with the integration of each module confirm their complementary roles in enhancing local perception, global modeling, and multi-scale feature fusion.

Table 4 presents the comparative results for different placement positions and connection orders in the encoder. When only a single module is introduced, both V1 and V2 outperform the baseline V0, indicating that either the local detail modeling of MobileViT or the window-based attention of Swin can yield effective performance gains. When MobileViT is deployed at different hierarchical levels (V3–V5), it can be observed that introducing it too early at shallow layers does not consistently produce benefits and tends to cause greater performance fluctuations. When MobileViT operates on deeper semantic layers, the resulting improvements are more stable and are more easily complemented by subsequent global relationship modeling. The final full configuration V6 achieves the best performance across all three metrics, which is consistent with its pathway design: local textures and small-object cues are first enhanced, followed by cross-region semantic association through Swin. When the order is reversed to Swin→MobileViT (V7), performance degrades markedly, demonstrating that the gains do not stem from merely “stacking modules,” but rather from more appropriate placement and cooperative integration.

**Table 4:** Location and Sequential Ablation Results for the Potsdam Dataset (V0–V7).

Variant	MobileViT stage	Swin stage	Order	mIoU (%)	mF1 (%)	OA (%)
V0	–	–	–	81.28	89.45	91.33
V1	S4	–	MobileViT only	84.57	91.54	92.06
V2	–	S4	Swin only	85.45	92.04	92.05
V3	S1	S4	MobileViT → Swin	85.44	92.04	92.08
V4	S2	S4	MobileViT → Swin	84.16	91.29	91.51
V5	S3	S4	MobileViT → Swin	85.23	91.91	91.96
V6 (RTAS-Net)	S4	S4	MobileViT → Swin	**87.10**	**93.20**	**92.60**
V7	S4	S4	Swin → MobileViT	83.97	91.19	91.63

### Visualization analysis on the potsdam dataset

**Fig 8:**
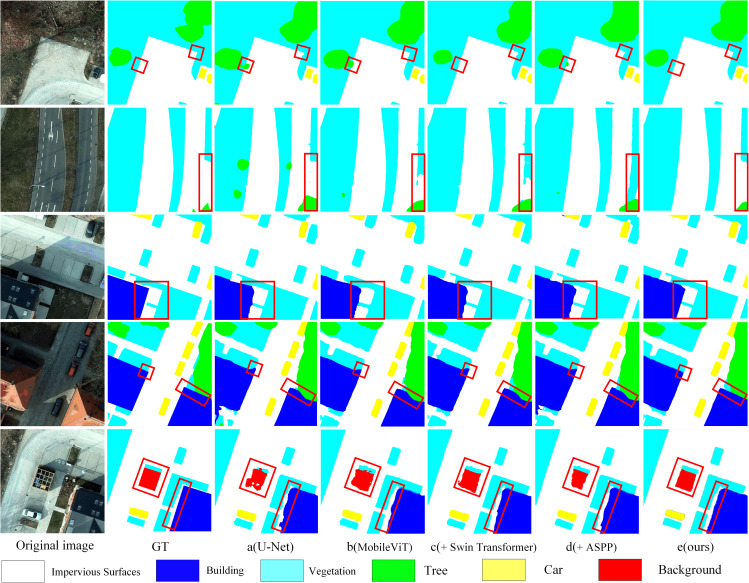
Visual Comparison of Segmentation Results from Different Models on the Potsdam Dataset (Red Boxes Highlight Key Comparison Areas).

To intuitively demonstrate the performance of the proposed model in remote sensing semantic segmentation, several representative samples from the Potsdam validation set are selected for qualitative visualization. In Fig 8, each row displays the original remote sensing image, the corresponding ground truth labels, and the segmentation outputs under different module configurations. These include the baseline model and the progressive integration of the MobileViT block, Swin Transformer, ASPP, and mini-ASPP modules. The side-by-side comparisons clearly highlight the individual contributions of each architectural component, visually demonstrating how each module enhances segmentation accuracy—particularly in boundary delineation, small object recognition, and multi-scale consistency.

As shown in Fig 8, the baseline model is more prone to fragmentation, missed detections, and boundary overflow at small objects and complex boundaries, with typical cases including vehicles being submerged by background and jagged misclassifications at the interfaces between roads and impervious surfaces. After incorporating MobileViT, the contours of small objects become more complete and the recall of vehicles and narrow linear structures is improved, with such changes being more evident in shadowed or texture-similar regions, indicating that enhancing local texture and neighborhood details helps alleviate appearance variations caused by illumination changes. With the further introduction of the Swin Transformer, the regional consistency of large-area categories (e. g., roads, impervious surfaces, and low vegetation) becomes more stable and the discontinuities across block-like regions are reduced. In occluded or locally missing areas, the predictions tend to maintain overall coherence, reflecting the compensatory effect of window-based attention on spatial context. The contributions of ASPP and mini-ASPP are mainly manifested in boundary and scale-mixed regions: the edges near tree–building interfaces and narrow structures adhere more closely to the annotations, and the alignment between shallow fine-scale information and high-level semantics before decoding is more sufficiently.

**Fig 9:**
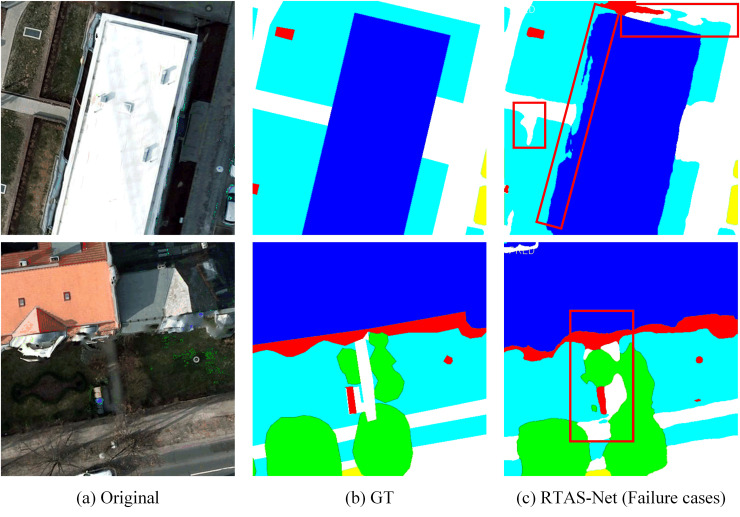
Typical failure cases on the Potsdam dataset.

To more comprehensively analyze the limitations of the model, Fig 9 presents two representative failure cases on the Potsdam dataset. From the first case, it can be observed that at the boundary between large-scale buildings and impervious surfaces, RTAS-Net still yields somewhat incomplete delineation of building edges, with some areas being misclassified as impervious surface. The second case shows that when roads are occluded by objects such as trees, the category predictions within the occluded regions exhibit pronounced confusion, which disrupts the continuity of the road structure. These observations indicate that, although RTAS-Net improves overall segmentation accuracy and structural consistency, it can still be affected by local texture interference and contextual uncertainty in regions with severe occlusion and ambiguous class boundaries.

### Ablation study on the vaihingen dataset

To further validate the effectiveness and generalizability of the RTAS-Net modular design across diverse remote sensing scenarios, ablation experiments were also conducted on the ISPRS Vaihingen dataset. Starting from a baseline U-Net architecture, key modules were incrementally integrated to progressively construct the full RTAS-Net model. The objective of this experiment is to quantitatively evaluate the individual contributions of each module to both category-specific segmentation accuracy and overall model performance, thereby confirming their effectiveness under varying spatial structures and scene complexities. Table 5 presents the performance of different module combinations on the Vaihingen dataset, evaluated using four metrics: mIoU, mF1, OA, and Params.

**Table 5:** Performance Comparison of Different Module Combinations (Vaihingen).

Model	Structural description	mIoU (%)	mF1 (%)	OA (%)	Params (M)
A	U-Net baseline	91.14	95.33	95.94	43.93
B	A + MobileViT	91.34	95.44	95.98	51.66
C	B + Swin Transformer	91.44	95.49	96.06	53.89
D	C + ASPP	91.80	95.69	96.28	69.96
E	D + mini-ASPP (ours)	**92.09**	**95.85**	**96.31**	**100.85**

As shown in Table 5, the model‘s performance improves incrementally with the progressive addition of modules, clearly demonstrating the complementary nature of each component. To further illustrate the semantic segmentation effectiveness of different architectural configurations on the Vaihingen dataset, a radar chart is presented in Fig 9. The chart depicts the IoU scores for six representative categories—background, cars, trees, low vegetation, buildings, and road surfaces—across five model configurations, from Model A (baseline U-Net) to Model E (full RTAS-Net). This visualization provides an intuitive understanding of how each module contributes to class-level segmentation performance under varying scene complexities.

**Fig 10:**
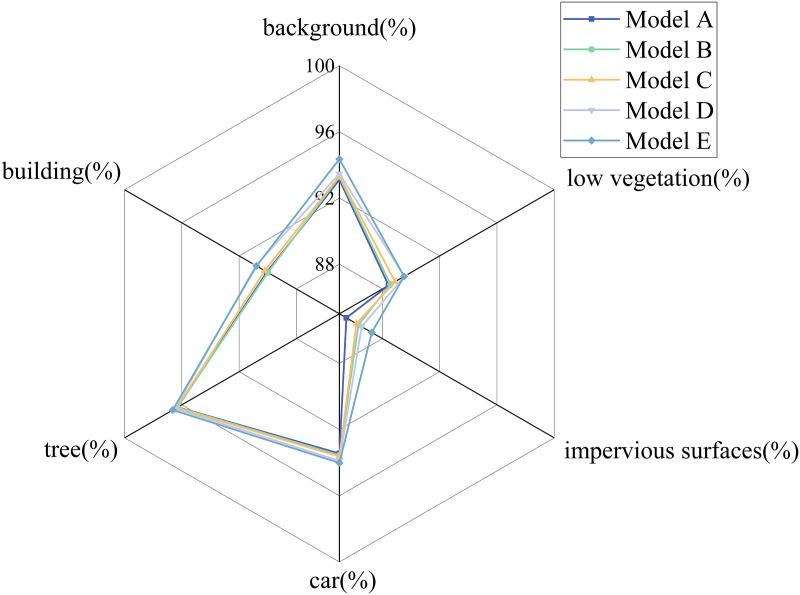
Radar Chart of Per-Class IoU for Ablation Models on the Vaihingen Dataset.

As shown in Fig 10, the complete RTAS-Net architecture achieves high segmentation accuracy across all evaluated categories, with particularly notable improvements in the background and low vegetation classes. These results underscore the effectiveness of modules such as the Swin Transformer and ASPP in enhancing the model‘s capacity to capture large-scale objects and represent complex boundary structures. Meanwhile, the inclusion of the MobileViT module significantly improves the detection of small objects, such as vehicles, further enhancing the model‘s fine-grained feature representation and cross-scene generalization capability.

Table 6 reports the effects of different placement positions and connection orders on the Vaihingen dataset. Compared with the baseline V0, introducing MobileViT alone (V1) yields almost no improvement, whereas introducing Swin alone (V2) leads to a more pronounced gain, indicating that global or cross-region semantic association is more critical for performance in Vaihingen-type scenes dominated by large-area land-cover classes with stronger regional consistency. When MobileViT is deployed at different hierarchical levels (V3–V5), the overall performance still exhibits mainly stable but limited gains, and early insertion at shallow layers does not show additional advantages, suggesting that the primary bottleneck of this dataset lies not in local texture enhancement but in maintaining cross-region semantic consistency and boundary continuity. The complete configuration V6 achieves the best results, demonstrating that MobileViT remains valuable as a complementary detail modeling component on top of the global associations provided by Swin. However, when the order is reversed to Swin→MobileViT (V7), the metrics decline, further indicating that the mode of module cooperation affects the effectiveness of information flow and that a well-designed pathway is more crucial than simply replacing or stacking components.

**Table 6:** Location and Sequential Ablation Results for the Vaihingen Dataset (V0–V7).

Variant	MobileViT stage	Swin stage	Order	mIoU (%)	mF1 (%)	OA (%)
V0	–	–	–	90.54	95.00	95.70
V1	S4	–	MobileViT only	90.54	95.00	95.67
V2	–	S4	Swin only	91.14	95.33	95.94
V3	S1	S4	MobileViT → Swin	90.20	94.81	95.73
V4	S2	S4	MobileViT → Swin	90.81	95.15	95.72
V5	S3	S4	MobileViT → Swin	90.43	94.94	95.70
V6 (RTAS-Net)	S4	S4	MobileViT → Swin	**92.09**	**95.85**	**96.31**
V7	S4	S4	Swin → MobileViT	91.34	95.44	95.98

### Ablation study on the vaihingen dataset

To further validate the specific impact of each module combination on segmentation performance from a visual perspective, representative image samples were selected from the Vaihingen dataset. These include the original aerial images, the corresponding ground truth annotations, and the predicted segmentation results from Model A to Model E. In Fig 10, the visual comparison emphasizes key regions—such as intersections between vehicles, roads, and buildings—highlighted with red bounding boxes. This layout allows for a clear and intuitive assessment of each model‘s ability to handle complex spatial structures and accurately segment small objects.

**Fig 11:**
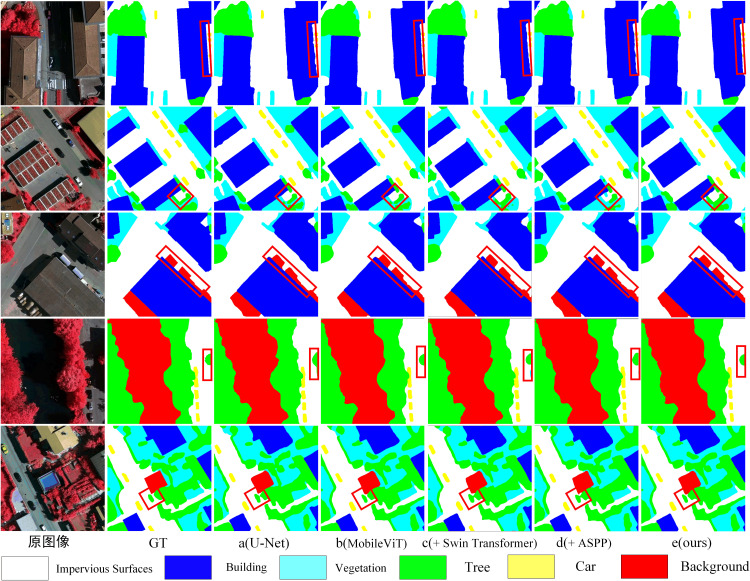
Visual Comparison of Segmentation Results from Different Models on the Vaihingen Dataset (Red Boxes Highlight Key Comparison Areas).

As shown in Fig 11, the baseline model is more prone to missing small objects such as vehicles, and the boundaries of buildings and roads are locally discontinuous. After incorporating MobileViT, the shapes of small objects and thin structures become clearer and boundary overflow is reduced. In regions with pronounced shadows or illumination variations, the model exhibits more stable discrimination of the same land-cover classes, indicating that fine-grained texture enhancement mitigates the impact of lighting differences on local classification. With the introduction of the Swin Transformer, the overall consistency of large-scale categories such as roads and buildings is further improved, and cross-region misclassification and fragmentation are reduced. In areas occluded by tree canopies or with locally missing textures, the predictions tend to preserve structural continuity, demonstrating the compensatory effect of contextual modeling in occlusion scenarios. With the subsequent integration of ASPP and mini-ASPP, multi-scale contextual information and shallow fine-scale features are more fully expressed at boundaries, the geometric contours of land-cover interfaces adhere more closely to the annotations, and the overall results become more regular.

**Fig 12:**
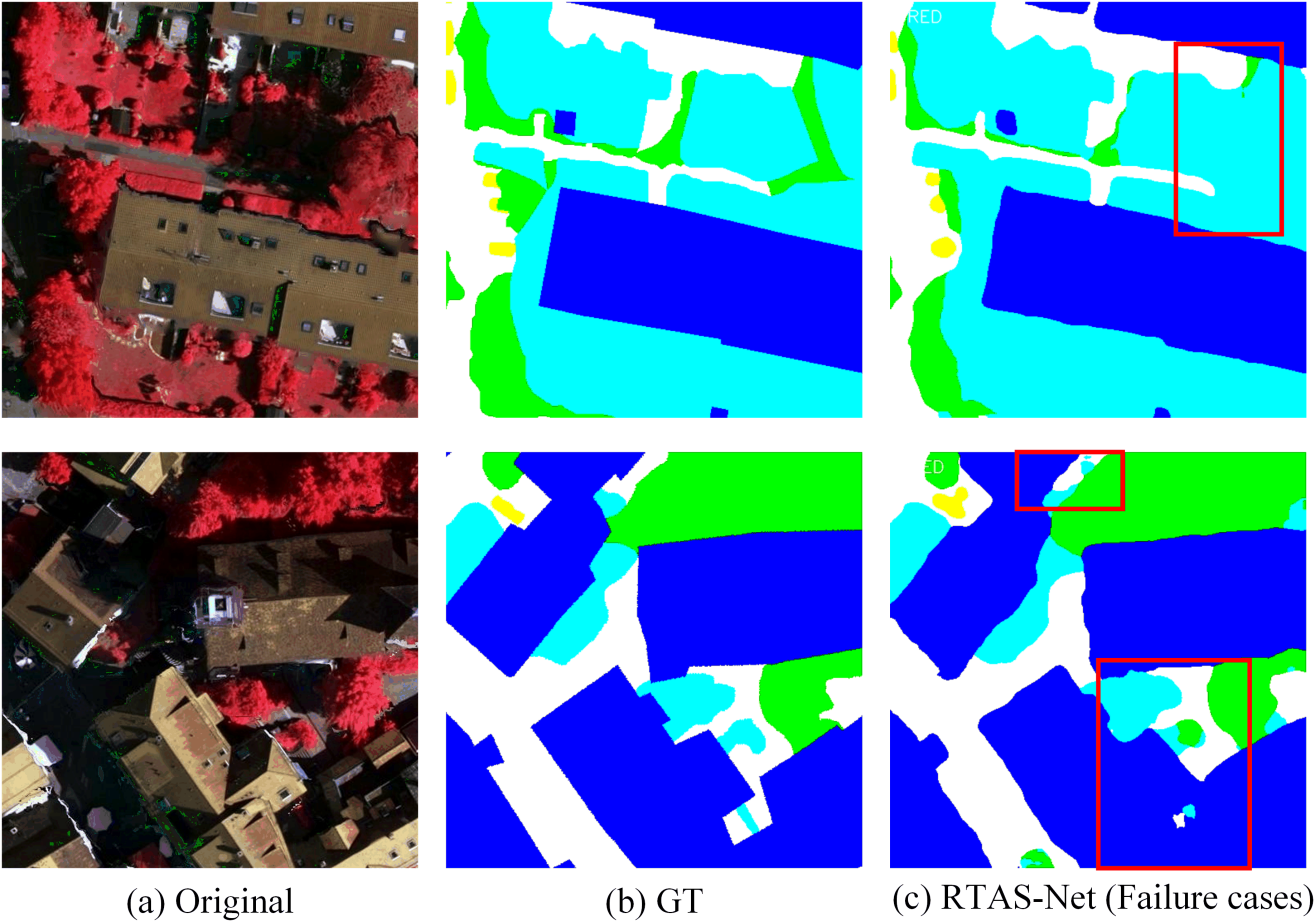
Typical failure cases on the Vaihingen dataset.

Fig 12 presents two representative failure cases of RTAS-Net on the Vaihingen dataset. From the first case, it can be observed that in regions at the interface between roads and low vegetation, the model still produces unstable delineation of the continuity of slender road structures, with some local areas being eroded by the vegetation class. The second case indicates that in scenes where buildings and trees are interspersed, small-scale regions are susceptible to interference from the textures of adjacent classes, leading to fragmented predictions and class confusion. These results suggest that in high-density urban scenarios, when strong occlusion or blurred boundaries exist between objects, relying solely on multi-scale context and window-based attention mechanisms is still insufficient to fully eliminate local uncertainty.

### Cross-dataset verification and hyperparameter sensitivity

The quantitative results and visual comparisons on the first two datasets have already revealed two consistent phenomena: small objects are more prone to missed detections, and complex boundaries are more susceptible to adhesion and fragmentation. This section introduces LoveDA, a public remote sensing segmentation benchmark, to examine the stability of the model under different data styles using more complex scenes and richer class distributions, while also observing the reproducibility of the training configuration under cross-dataset conditions. The experimental settings are kept consistent with the previous ones, with no additional changes to the input resolution or training pipeline, and the evaluation is conducted using mIoU, mF1, and OA. To ensure fair comparison, the incremental structural design from A to E is still adopted, the training strategy is kept unchanged, and only the module combinations and the loss weight parameters discussed later are modified.

Table 7 reports the results of different module combinations on the LoveDA dataset. It can be observed that the baseline structure A achieves an mIoU of 63.99% on LoveDA. After incorporating MobileViT (B), the metrics exhibit a slight increase, with mIoU rising to 64.17% and mF1 and OA improving accordingly, indicating that local texture enhancement remains effective on this dataset. With the further integration of the Swin Transformer (C), mIoU continues to increase to 64.18%, showing only a marginal change, while mF1 still improves, suggesting that the benefits of long-range dependency modeling are more reflected in the stability of class discrimination. After adding ASPP (D), the improvement becomes more pronounced, with mIoU reaching 64.73% and mF1 increasing to 78.29%, which is consistent with the more complex scale variations characteristic of LoveDA scenes. Finally, with the inclusion of mini-ASPP (E), mIoU is further increased to 64.82%, achieving the best result in this group, and mF1 simultaneously reaches 78.38%. The overall trend indicates that on a dataset with more complex distributions such as LoveDA, the gains brought by multi-scale context aggregation and shallow fine-scale alignment are more stable. Moreover, the benefits of module combinations do not rely on any “incidental characteristics” of a specific dataset, and the results remain consistent with the observations on the previous two datasets.

**Table 7:** Performance Comparison of Different Module Combinations (LoveDA).

Model	Structural description	mIoU (%)	mF1 (%)	OA (%)
A	U-Net baseline	63.99	77.76	78.18
B	A+MobileViT	64.17	77.79	78.40
C	B+Swin Transformer	64.18	77.90	78.18
D	C+ASPP	64.73	78.29	78.75
E	D+mini-ASPP(ours)	**64.82**	**78.38**	**78.58**

On this basis, Table 8 further analyzes the influence of the edge-term weight *λ* in the loss function. When the edge term is disabled (λ=0), the mIoU and mF1 of the model are relatively low. After introducing the edge term, the overall metrics are improved, and the best performance is achieved at λ=0.4. When the weight is further increased to 0.6, the accuracy declines, indicating that an overly strong edge constraint competes with the learning of regional consistency, causing the model to focus more on local contours at the expense of stable region-level discrimination.

**Table 8:** Sensitivity to edge-weight *λ* in the loss function on LoveDA.

Metric \ *λ*	0 (Off)	0.2	0.4 (default)	0.6
mIoU (%)	64.22	64.73	**64.82**	64.36
mF1 (%)	77.87	78.28	**78.38**	77.98
OA (%)	78.42	78.50	**78.58**	78.30

### Computational efficiency and resource overhead analysis

After the ablation experiments have verified the accuracy gains brought by each module, this section further evaluates the efficiency of different architectures from the perspective of computational overhead. Statistics are collected under the same input setting (512×512 patches) and hardware environment as in the previous sections, using parameter count (Params), computational cost (FLOPs), peak GPU memory (Peak Memory), and single-image inference latency (Latency) as the main metrics, and providing inference throughput (Inference FPS, images/s) as an intuitive reference.

As shown in Table 9, the incremental module combinations (A–E) exhibit a clear pattern of increasing computational overhead. Model A has the lowest parameter count, FLOPs, and memory consumption. Introducing MobileViT leads to the main increase in parameters and computation, accompanied by the expected rise in inference latency and reduction in throughput. When Swin Transformer and ASPP are added on top of Model B, the growth in parameter count is relatively moderate, whereas peak memory usage and latency still increase due to the inclusion of global modeling and multi-scale branches. The complete architecture, Model E, reaches the highest parameter count and FLOPs, but under the 512×512 input setting it still maintains stable millisecond-level latency and practical inference throughput. Further examination of the position- and order-related variants (V0–V7) shows that most variants have similar parameter counts and FLOPs to their corresponding module combinations. The differences are mainly reflected in fluctuations of peak memory and latency, indicating that efficiency is more strongly affected by module placement and feature resolution than by the mere presence or absence of specific modules.

**Table 9:** Computational efficiency of module-combination models (A–E) and placement/order variants (V0–V7).

Model	params (M)	FLOPs (G)	Peak Mem (GB)	Latency (ms/img)	Inference FPS (img/s)
A	43.93	79.68	0.28	8.16	122.55
B	50.87	110.30	0.404	10.30	97.09
C	51.66	111.16	0.596	10.67	93.72
D	53.89	113.38	0.798	10.88	91.91
E (RTAS-Net)	**100.85**	**228.78**	**1.19**	**19.39**	**51.57**
V0	70.16	197.30	1.449	17.04	58.69
V1	100.06	227.92	1.565	19.18	52.14
V2	70.95	198.16	1.451	17.19	58.17
V3	101.11	229.06	1.569	19.56	51.12
V4	101.37	229.32	0.610	20.67	48.38
V5	100.85	228.78	0.987	19.48	51.33
V6 (E)	**100.85**	**228.78**	**1.19**	**19.39**	**51.57**
V7	101.11	229.32	0.988	19.47	51.36

## Conclusion

To address the typical challenges of large-scale variation, blurred boundaries, and complex semantic class distributions in remote sensing image semantic segmentation, this paper proposes a multi-structure integrated model, RTAS-Net, based on cooperative fusion paths and complementary module design. The network adopts ResNet50 as the backbone and introduces MobileViT at the high-semantic encoding stage to enhance small-object representation and local texture modeling, while incorporating the window-based attention mechanism of the Swin Transformer to capture cross-region semantic associations. In addition, ASPP is employed at the end of the encoder to aggregate multi-scale context, and mini-ASPP is embedded into each skip connection to perform fine-scale pre-fusion of shallow high-resolution features, thereby alleviating semantic mismatch between shallow and deep features before decoding, while a composite loss of Dice Loss, Focal Loss, and an edge-guided term is used during training to stabilize optimization under class imbalance and boundary ambiguity.

The experimental section systematically evaluates the model performance on ISPRS Potsdam and Vaihingen and further explains the sources of performance gains through incremental module combinations and ablations on placement and ordering. Different modules exhibit complementary effects in terms of small-object representation, regional consistency, and multi-scale contextual modeling, and appropriate injection levels and connection orders have a significant impact on the final performance. In addition to accuracy metrics, this work reports efficiency results including parameter count, computational cost, memory consumption, and inference latency to characterize the trade-off between accuracy gains and resource overhead. Cross-dataset verification and sensitivity analysis of the edge loss weight are conducted on the LoveDA dataset, and the results demonstrate that the model maintains stable improvements under more complex scenes and pronounced style variations, while the training configuration shows a certain degree of robustness to hyperparameter perturbations. RTAS-Net exhibits a clear trade-off among accuracy, stability, and computational cost, providing a reproducible and structured integration solution for high-resolution remote sensing semantic segmentation.
